# Self-care competence in the administration of insulin in older people aged 70 or over^1^

**DOI:** 10.1590/1518-8345.2080.2943

**Published:** 2017-10-30

**Authors:** Mayara Sousa Vianna, Patrícia Aparecida Barbosa Silva, Cíntia Vieira do Nascimento, Sônia Maria Soares

**Affiliations:** 2 MSc, RN, Departamento de Atenção à Saúde do Trabalhador, Universidade Federal de Minas Gerais, Belo Horizonte, MG, Brazil.; 3 PhD.; 4 Doctoral student, Escola de Enfermagem, Universidade Federal de Minas Gerais, Belo Horizonte, MG, Brazil. RN, Hospital Júlia Kubitschek, Fundação Hospitalar do Estado de Minas Gerais, Belo Horizonte, MG, Brazil.; 5 PhD, Associate Professor, Escola de Enfermagem, Universidade Federal de Minas Gerais, Belo Horizonte, MG, Brazil.

**Keywords:** Insulin, Elderly, Self-administration, Diabetes Mellitus, Nursing

## Abstract

**Objective:**

to analyze the self-care competence in the administration of insulin performed by older people aged 70 or over.

**Method:**

cross-sectional study carried out with 148 older people aged 70 or over, who performed self-administration of insulin. Data collection was carried out using a structured questionnaire and an adapted guide for the application of the Scale to Identify Self-Care Competence of Patients with Diabetes, at the participants’ home. Data analysis included descriptive and inferential statistical tests, with forward logistic regression.

**Results:**

the prevalence of self-care competence in the administration of insulin was 35.1%. Handwashing error was the most frequent in self-administration of insulin. Self-care competence was negatively associated with retirees and positively associated with senior patients who performed capillary blood glucose monitoring and skin pinching during insulin application.

**Conclusion:**

there was low self-care competence and it was associated with both the sociodemographic and the clinical characteristics with regard to self-application of insulin by the older people.

## Introduction

Population aging and the influence of risk factors, such as obesity, sedentary lifestyle and inadequate food consumption, have contributed to the increase in the prevalence of chronic non-communicable diseases, especially Diabetes *Mellitus* (DM)^1^. In 2015, there were an estimated 415 million (8.8%) diabetic people worldwide, and people with this disease are expected to increase to 642 million in the next two decades^2^. In line with the global trend, the prevalence of DM in the Brazilian scenario increased from 3.6% (95%CI: 3.3-3.8) in the 1990s to 6.1% (95%CI: 5.6-6.7) in 2015^1^, increasing with aging (19.8% in individuals aged 65 years or over)^3^. This chronic condition was responsible for 62,466 deaths in the country and 1,015 wasted years, disability-adjusted life-years per 100,000 inhabitants^1^.

In the literature, it is observed that the geriatric population is more susceptible to micro and macrovascular complications due to a decrease in physiological reserve capacity, which leads to functional decline^4^. In addition, older adults aged 75 years or over are at an increased risk for death due to hyperglycemic events, as well as hospital readmissions due to hypoglycemic episodes, compared to younger age groups^5^.

Among the therapeutic alternatives for metabolic control, there is the insulin therapy. Recent data from a series of studies of the *National Health and Nutrition Examination Surveys* (NHANES) program, which included 4,947 diabetic participants (≥20 years) in the United States of America, showed a prevalence of insulin therapy of 29.1% (95%CI: 26.7-31.5) in the period from 2005 to 2012. Considering only older people (≥65 years), this percentage was similar to that found for adults (27.8-29.8%, p-value=0.391), and 15.7% also used oral antidiabetic agents^6^.

According to current guidelines in the country, insulin therapy is indicated for people with type 2 DM (DM2), when changes in lifestyle and use of metformin are not sufficient for glycemic control after one month of therapy. Insulin therapy is also indicated when glycosylated hemoglobin (HbA1c) values are above 8.5%, even after the use of the maximum dose of metformin and persistent symptoms of hyperglycemia, or when serum glucose levels are >300 milligrams per deciliter (mg/dL)^7^.

Self-administration of insulin requires from the user the mastery of the cognitive and psychomotor skills that constitute the learning of different procedures, including storage, transportation, preparation of the solution and application, as well as handling of syringes, needles or injection pens^7^. Therefore, proper use of insulin at home requires training, changes in daily life, discipline, availability for learning and dedication and interest to perform self-care. The types of insulin and its forms of use are varied and, depending on the number of daily applications and the effect of the dose and type of insulin used, the responsibility of the patient in the control of this chronic condition increases^8^.

The self-administration of insulin requires the individual to have competence for such activity. Competence is the ability or capacity of an individual to perform certain activities, make decisions in the context of certain events, facts or circumstances. Self-care competence is the power of individuals to perform their own care in order to meet their particular needs. Such competence accompanies the development of individuals, beginning in childhood, reaching the highest degree of development in adulthood and decreasing in old age^9^.

Inaccuracies in the self-administration of insulin may adversely influence metabolic control, accelerate the progression of diabetes complications, and lead to discontinuation of treatment^10^. In addition, this situation is even more worrying among older people due to other factors added, such as visual deficit and cognitive and motor impairment, which make it difficult the self-administration of insulin and the Capillary Glycemia (CG) monitoring^11^.

The epidemiological aspects of DM have been extensively studied^1^ ^,^ ^12^, whereas there is a lack of information on self-administration of insulin, especially in older people. Previous studies at national level have identified frequent errors in the preparation and administration of insulin^10^, as well as improper disposal of residues generated by the administration of insulin at home^13^. This reality is not restricted only to Brazil, being also highlighted in studies at international level^14^ ^-^ ^16^.

Therefore, in order to enhance the knowledge on the subject, the objective of this study was to analyze the self-care competence in the administration of insulin performed by older people aged 70 or over with DM.

## Method

This cross-sectional study was carried out in the Northwest Sanitary District of the city of Belo Horizonte, MG, Brazil, with older people aged 70 or over, who performed self-administration of insulin.

The older people who met the criteria mentioned above were considered as potential participants. Older people whose electronic medical records did not contain telephone contact information (n=283), telephone contact without success (n=213), change of address (n=25), death (n=46) and refusal to participate (n=82) were excluded from the sample.

The study population consisted of individuals aged 70 years or over, enrolled in the Health System Management Network (Sisrede), who acquired insulin at the Health Centers (HC) of the Northwest District, from January 2014 to June 2015, no matter if they had DM type 1 or DM type 2.

For the sample size calculation, it was considered population size (N) equal to 1.193, proportion (P) of 27.54%^17^ for the self-administration of insulin, 95% confidence interval (expressed as Z^2^α/2=1.96) and a maximum tolerable error (ε) of 0.05^18^. The total sample consisted of 245 individuals.

A team composed of one nurse and four nursing students carried out data collection, in three stages. In the first stage, the team responsible for data collection was instructed to systematize data collection, in addition to enhance the knowledge about DM, insulin use and research methodology. A pilot study with five individuals was also carried out in order to determine the appropriate adjustments in the collection instruments, the estimated collection duration and other schedules.

In the second stage, a search for the names of older people, identified according to the HC, was performed in the Sisrede electronic medical record, in order to identify the Community Health Agents (CHA), the Family Health Teams, the telephone contact and the addresses of the older people. In this way, contact with the managers of the participating HC was made to explain the project. Subsequently, telephone contact was made with each of the older people or their caregivers to identify those who performed self-administration of insulin and to schedule a home visit. All older people who performed self-administration of insulin were selected in order to schedule a home visit, in up to three attempts.

In the third stage, two data collection instruments were used: the questionnaire and the adapted guide for the application of the Scale to Identify Self-Care Competence of Patients with Diabetes (ECDAC)^19^. This scale was developed and validated by a nurse in 1982, at the Federal University of Santa Catarina (UFSC), based on the concept of self-care competence, according to the Dorothea Orem’s theory^9^. In order to measure the reliability of the instrument, it was applied to 81 adults with DM, at their homes or at health institutions, and an alpha coefficient of 0.92 was obtained for the 27 items of the questionnaire^19^.

The first part of the questionnaire includes the sociodemographic and clinical data of the older people, as well as the register of alterations of the physical, mental and motivational capacities, which can affect self-care competence. The second part concerns the instrument to support the application of the Mini-Mental State Examination (MMSE). The third part of the instrument was used to register the errors found in the self-administration of insulin performed by the older people, with 20 previously defined errors as references, including the pre-application, application and post-application phases. For this purpose, self-administration of insulin performed by the older people at home was monitored and the errors and successes found were registered. Subsequently, the older person was instructed on the correct practices for self-application of insulin. Those who presented errors that could lead to the application of incorrect doses of insulin were immediately instructed on the proper way, and their relatives were informed about the risks of the self-application of insulin performed by the older people.

In this study, self-care competence was the dependent variable (up to 77 points for unsatisfactory self-care competence and 78 points or more for satisfactory self-care competence). The independent variables were the sociodemographic variables (age group, place of birth, sex, marital status, retirement, occupation, education level and living arrangement), clinical variables (length of DM diagnosis, family history, sedentary lifestyle, smoking, length of insulin use, type of insulin, use of injection pens, use of syringe, needle reuse, CG monitoring performance, CG mapping, current values of fasting glycemia levels and HbA1c values registered in the electronic medical record, comorbidities, number of medications, MMSE results), variables related to limitations for self-care (physical, mental and motivational limitations) and variables related to the self-application of insulin performed by older people.

The collected data were inserted into the database built using the *Statistical Package for the Social Sciences* (SPSS), version 20.0, which facilitated the analysis of the results.

The sample size was equal to 148, unlike the prior estimate of 245, due to difficulties in terms of telephone contact and agreement to schedule the home visits by telephone, making it necessary to calculate the sample power. The significance level was set at 5% and the null hypothesized proportion was 27.54%. Maximum likelihood estimation was considered for the alternative hypothesis, that is, the estimate for the proportion found in the sample was 45.8%, resulting in a power of 0.99^20^ ^-^ ^21^.

The descriptive analysis for the variables was performed using frequency distribution tables. Continuous variables were expressed as mean±Standard Deviation (SD), for variables with parametric distribution, and as median with Interquartile Range (IQR Q3-Q1), for variables with non-parametric distribution. Categorical variables were expressed as proportions or percentages.

Continuous variables were tested for normal distribution using Kolmogorov-Smirnov test and Spearman’s Rho correlation analysis was calculated between self-care competence scores and physical, mental and motivational limitations.

In the univariate analysis, categorical variables were compared using Pearson Chi-square test (x^2^) or Fisher’s exact test. For statistical modeling, a critical value of p≤0.20 was used for entry into the multivariate model. A logistic regression model was developed using the *forward* method to evaluate the direction and magnitude of the associations of each independent variable in relation to the response variable (self-care competence). For this analysis, p<0.05 was considered as statistically significant. The achieved values were expressed as *Odds Ratio* (OR) and their respective 95% confidence intervals. The final model fit was assessed using *goodness-of-fit test*.

The Research Ethics Committee approved this study under Protocol number 1.004.545, in accordance with Resolution 466/2012 of the National Health Council^22^.

## Results

The profile of older people was characterized as a predominantly female population (64.2%), coming from the interior of the state of Minas Gerais (61.5%), without a spouse (54.7%), with a level of education equal to or less than four years of study (59.5%). The predominant age group was from 70 to 79 years (73.6%). It was observed that 8.8% of the older people still worked in the period in which the study was carried out, whereas 78.4% were retired. Regarding the living arrangement, 14.2% of the study population lived alone.

The majority of older people reported having Systemic Arterial Hypertension (SAH) (93.9%), dyslipidemia (64.9%) and diabetic retinopathy (31.8%). It was found that 72.3% of older people used five to ten medications. In addition, 65.5% of individuals reported a sedentary lifestyle and 7.4% smoked.

Regarding DM, 76.8% had the disease for more than ten years and 58.9% used insulin for less than 10 years. Family history of DM was observed in 63.5%.

The use of syringes for the application of insulin was frequent among older people in 90.5% of the cases, and 70.3% reused the needle of the syringe or the needle of the injection pen from two to eight times. Only 67.6% of older people often perform CG monitoring, and only 37.2% had the capillary blood glycemia values registered in a graphic format. Regarding the results of the laboratory tests, fasting blood glycemia levels were higher than 130 mg/dL in 61.6% and 52.1% had HbA1c levels equal to or greater than 8%.

The evaluation of errors (non performed or improperly performed) in the self-application of insulin showed a higher prevalence of errors during handwashing (87.2%), aspiration of air into the syringe and injection into the insulin vial (74.3%) and during the disposal of sharp objects into a hard flask (73.1%).

The following are the frequencies of procedures properly or improperly performed, as well as the non-performance of the procedures by older people (Table 1).

**Table 1:** Frequency of errors and successes in the self-administration of insulin. Northwest Sanitary District. Belo Horizonte, MG, Brazil, 2015 (N=148)

**Variables**		**Performance of the procedure**										
		**Yes**									**No**	
		**Properly**			**Improperly**			**Total**			**n**	**%**
		**n**	**%**		**n**	**%**		**N**	**%**			
**Pre-application**												
	**Handwashing**	**19**	**90.5**		**2**	**9.5**		**21**	**14.2**		**127**	**85.8**
	**Withdrawal of insulin from the refrigerator** ^**(n=141)**^	**39**	**59.1**		**27**	**40.9**		**66**	**46.8**		**75**	**53.2**
	**Organization of the material for use** ^**(n=147)**^	**102**	**87.2**		**15**	**12.8**		**117**	**79.6**		**30**	**20.4**
	**Differentiation of insulin types** ^**(n=42)**^	**36**	**97.3**		**1**	**2.7**		**37**	**88.1**		**5**	**11.9**
**Application technique**												
	**Rotation of application sites**	**76**	**82.6**		**16**	**17.4**		**92**	**62.2**		**56**	**37.8**
	**Application site delimitation**	**109**	**91.6**		**10**	**8.4**		**119**	**80.4**		**29**	**19.6**
	**Disinfection of the insulin vial** ^**(n=140)**^	**46**	**88.5**		**6**	**11.5**		**52**	**37.1**		**88**	**62.9**
	**Shaking NPH insulin** ^**(n=141)**^	**80**	**80.0**		**20**	**20.0**		**100**	**70.9**		**41**	**29.1**
	**Aspiration of regular insulin before NPH** ^**(n=28)**^	**19**	**95.0**		**1**	**5.0**		**20**	**71.4**		**8**	**28.6**
	**Aspiration of the prescribed dose of insulin** ^**(n=143)**^	**120**	**100.0**		**0**	**0.0**		**120**	**83.9**		**23**	**16.1**
	**Aspiration of air into the syringe and injection** ^**(n=140)**^	**36**	**100.0**		**0**	**0.0**		**36**	**25.7**		**104**	**74.3**
	**Removal of air bubbles** ^**(n=125)**^	**46**	**76.7**		**14**	**23.3**		**60**	**48.0**		**65**	**52.0**
	**Skin pinching for application**	**99**	**94.3**		**6**	**5.7**		**105**	**70.9**		**43**	**29.1**
	**90º angulation for needle insertion**	**105**	**100.0**		**0**	**0.0**		**105**	**70.9**		**43**	**29.1**
	**Subcutaneous needle insertion**	**127**	**93.4**		**9**	**6.6**		**136**	**91.9**		**12**	**8.1**
	**Wait time to withdraw of needle**	**44**	**89.8**		**5**	**10.2**		**49**	**33.1**		**99**	**66.9**
**Post-application**												
	**Care in reusing the needle** ^**(n=142)**^	**106**	**85.5**		**18**	**14.5**		**124**	**87.3**		**18**	**12.7**
	**Disposal of sharps into a hard flask** ^**(n=145)**^	**39**	**50.0**		**39**	**50.0**		**78**	**53.8**		**67**	**46.2**
	**Bleeding: pressure on the application site** ^**(n=127)**^	**42**	**68.9**		**19**	**31.1**		**61**	**48.0**		**66**	**52.0**
	**Storage of the opened insulin vial up to 30°C** ^**(n=147)**^	**71**	**59.7**		**48**	**40.3**		**119**	**81.0**		**28**	**19.0**

The analysis of the associations between self-care competence scores and physical, mental and motivational limitations showed a strong correlation only with respect to mental limitation (r=0.824), whereas motivational limitation showed a moderate correlation (r=0.545) and physical limitation showed a weak correlation (r=0.353).

The results of univariate analysis demonstrated a significant association between an adequate self-care competence and several factors such as: not being retired, use of two types of insulin, needle reuse from two to eight times, performance of CG monitoring, preserved mental state, proper delimitation of the insulin site application, correct performance of skin pinching for application and needle insertion for application at a 90º angle.

Regarding the multivariate model, there was a negative association between self-care competence and retirement, and a positive association between self-care competence and the performance of CG monitoring and the performance of skin pinching for insulin application, as shown in Table 2.

**Table 2:** Final logistic regression model with self-care competence as an outcome variable. North West Sanitary District. Belo Horizonte, MG, Brazil, 2015 (N=148)

**Variables**		OR*	**95%CI** ^**†**^	**P-value**
**Retirement**			**0.12-0.68**	**0.005**
	**No**	**1.00 (ref.)**		
	**Yes**	**0.29**		
**Skin pinching for application**			**1.68-10.05**	**0.002**
	**Properly/do not perform**	**1.00 (ref.)**		
	**Improperly**	**4.11**		
**Perform capillary glycemia**			**1.17-6.32**	**0.020**
	**No**	**1.00 (ref.)**		
	**Yes**	**2.72**		

*Odds ratio; **†**95%CI: 95% confidence interval

## Discussion

In this study, an adequate self-care competence in the administration of insulin was identified among participants. The results revelead the mental limitation as the indicator that most affected the self-care competence. Previous studies have shown that cognitive impairment increases the risk of nonadherence to diet, lack of glycemic monitoring, inadequate metabolic control, impaired quality of life^23^, severe hypoglycemia^24^, and difficulty performing complex self-care tasks, such as inadequate self-administration of insulin and inadequate dietary maintenance in terms of time and content. In this context, older people with cognitive impairment should be subject of individualized actions performed by health professionals, which require the participation of caregivers and family members and special attention to the detection of possible occurrence of hypoglycemia^5^.

An important finding of this study is the high percentage of participants with altered metabolic control, which may have been influenced by the inadequate self-care competence. A study carried out with diabetic patients undergoing insulin therapy for two years or more demonstrated a positive association between adequate glycemic control and confidence in the choice of insulin dosage, as well as a proper time interval between applications^15^. Inappropriate doses and applications of insulin may alter glycemic levels and cause a higher or lower rate of drug absorption.

Preprandial blood glucose readings in adults should not exceed 130 mg/dL and the desirable HbA1c level is up to 7%. In older people, the tolerable HbA1c level is 8%, due to their greater vulnerability to hypoglycemic episodes and high risk of falls^7^. International recommendations suggest that, for functionally dependent older people, the target for HbA1c levels ranges from 7 to 8% and, specifically, for fragile older people or those with mental illness, these levels may reach up to 8.5%^25^.

Motor and visual limitations are additional aggravating factors, which may compromise the insulin self-application technique, making it difficult to handle the application device accurately and therefore have an impact on the metabolic control^10^. It should be noted that one in three participants had diabetic retinopathy, and vision impairment or loss of vision may affect the self-administration of insulin performed by older people, since it is necessary to visualize the syringe’s graduation to ensure the right application dose and differentiate the types of insulin. It is important to note that the numbers indicating the graduation on the syringe are usually small, and sometimes with faint or blurred outlines, and the letters printed on the insulin vials indicating the insulin type and the expiration date are very small, which makes it difficult for older people to see.

In another respect, the high percentage of polypharmacy evidenced in this study is cause for concern, possibly due to the high burden of chronic diseases among long-lived individuals. A population-based study on Health, Well-Being and Aging (SABE), conducted in São Paulo, SP, Brazil, with people aged 60 years or over, found that individuals aged 75 years or over are 1.9 times more likely to have polypharmacy (OR: 1.9, 95% CI: 1.3-2.7, p-value: 0.001), in relation to younger age groups; and diabetes was the predictor variable showing the greatest strength of association with outcome (OR: 4.1, 95% CI: 2.2-7.5, p-value <0.001)^26^. The therapeutic complexity associated with multiple comorbidities can lead to an inappropriate use of medications by older people, making self-care more complex, especially in cases where cognitive alterations are present. In addition, polypharmacy increases the chances of drug interactions and side effects, which can lead to intoxications, potentiation of drug effects and increased risk of falls^5^.

It is worth mentioning that almost all older people with diabetes also had SAH, which probably requires the concomitant use of antihypertensives. Previous studies^27^ ^-^ ^28^ have demonstrated the influence of antihypertensive agents on glycemic control, pointing out the thiazide diuretics and beta-blockers as the pharmacological classes associated with insulin resistance and poor glycemic control.

Regarding the frequency of errors and successes in self-administration of insulin, handwashing was not performed by the majority of participants, a result that did not coincide with that of two other similar studies. One of these studies, conducted by phone contact, found that only 3.9% of participants did not perform handwashing^29^, and the other study, carried out through a semi-structured interview, found that 11.2% performed handwashing sometimes or never^30^. The recommendation is to wash hands with soap and water before preparing insulin^31^.

Regarding air aspiration in the syringe and injection into the insulin vial prior to aspiration of the content, most participants did not perform this procedure, a result similar to that found in other studies^29^ ^-^ ^30^. Air should be injected into the insulin vial in the amount corresponding to the dose to be drawn from the vial to prevent vacuum inside the vial^7^.

Regarding the disposal of sharps, approximately half of the older people discarded sharps in a hard flask and only half of them performed the procedure properly. Similar results were found in a recent study conducted in the city of Fortaleza, CE, Brazil, which showed that 57.1% of the participants discarded in an inappropriate manner the waste generated by the administration of insulin in the ordinary household waste, even though they had been instructed on the appropriate manner to perform the disposal of this material^13^. International studies^32^ ^-^ ^33^ also found high percentages of inappropriate waste disposal resulting from insulin therapy, and the possible reason for this finding was the unsatisfactory impact of continuing education on the safe disposal of sharps. As a consequence, there is an increased risk of contamination of the environment, transmission of pathogens and accidents with sharps, which demands the intensification of sanitary measures and greater commitment of health professionals in the guidance of the community^16^.

Considering the possible predictors of self-care competence, this study confirmed the multifactorial nature of the phenomenon in evidence, and self-care competence was associated with both the sociodemographic and clinical characteristics and the performance of self-administration of insulin by older people.

A negative association was found between retirement and self-care competence, which may be due to the time devoted to self-care over a lifetime. Since all older people were over 70 years, the mandatory retirement age, it can be inferred that older people who paid their national insurance contributions (INSS), and possibly worked outside the home throughout their lives, dedicated less attention to take care of DM than older people who worked at home and had more free time to take care of their health, as well as to activities to encourage self-care behaviors in DM management, such as participation in operative groups.

Regarding the effect of CG monitoring on self-care competence, a positive association was identified, ie, older people who performed CG monitoring regularly also showed an adequate self-care competence. This association was already expected, since one of the self-care activities of the insulin user is the CG monitoring, an important practice to monitor the individual and for the adequacy of the insulin dose required for the individualized treatment of DM.

A study conducted in the city of Botucatu, SP, Brazil, identified that only 9.7% of the users were competent to take actions based on the results of CG monitoring, at home. To make matters worse, health professionals underutilized the results of CG self-monitoring performed by users. The authors emphasize that CG self-monitoring at home allows the development of autonomy and decision-making skills in order to achieve an adequate glycemic control, and consequently, a reduction of the acute and chronic complications of the disease and the improvement of quality of life^34^.

Considering the positive association found between the performance of skin pinching and the self-care competence, it is understood that older people who perform skin pinching for the administration of insulin also showed self-care competence. Properly performing the skin pinching during insulin administration ensures effective absorption of insulin into the subcutaneous tissue and avoids the risk of hypoglycemia in cases of accidental intramuscular administration, as well as the extravasation of medication, pain, local reaction and elevation of the skin and hyperglycemia in case of intradermal administration^35^.

One of the essential ways of making the person with DM aware of the importance of self-care is through the implementation of educational practices, and the participation of the nurse in these practices is fundamental. To educate aiming at promoting self-care competence depends on the professional’s technical competence, as well as the patient’s willingness and interest^36^. The objective of this educational process is to provide strategies that promote behavior change, contributing to the improvement of the quality of life of the person with DM^37^.

Regarding the limitations of this study, the lack of references on the self-application of insulin specifically by the older people is highlighted, which made it difficult to develop the statistical calculations for the estimation of sample size and the calculation of the sample power. Since this is a cross-sectional study, it was not possible to establish a cause and effect relationship between the variables.

Other limitations should be considered, such as difficulty in telephonic contact with the older people due to incorrect or missing telephone numbers, lack of financial resources and short deadlines that did not allow search for the addresses without a prior telephone contact.

## Conclusion

Based on the results of this study, it was identified that handwashing, air aspiration in the syringe and injection in the insulin vial and disposal of sharps in a hard flask are the main errors in the self-application of insulin. Only the mental limitation showed a strong correlation with self-care competence. Retirement, performance of CG monitoring and skin pinching for insulin application were associated with the outcome variable.

In view of these results, it is considered essential the interdisciplinary care to the older person with DM through a special and individualized care, which must be provided to this population. In addition, identifying and analyzing errors and difficulties related to the self-care competence in the administration of insulin in older people aged 70 or over with DM, enables professionals to contribute in their area of knowledge and stimulate a comprehensive care focused on older people.
